# ‘For Want of a Nail’: developing a transparent approach to retroduction and early initial programme theory development in a realist evaluation of community end of life care services

**DOI:** 10.1080/13645579.2023.2184920

**Published:** 2023-03-12

**Authors:** Kathryn McEwan, Melissa Girling, Angela Bate, Joanne Atkinson, Amanda Clarke, Sonia Dalkin

**Affiliations:** a Dept of Nursing, Midwifery and Health, University of Northumbria at Newcastle, Newcastle Upon Tyne, United Kingdom; b Dept of Social Work, Education and Community Wellbeing, Faculty of Health & Life Sciences, University of Northumbria at Newcastle, Newcastle Upon Tyne, United Kingdom

**Keywords:** Realist evaluation, retroduction, end of life care, realist methodology, transitions theory

## Abstract

A crucial part of theory-driven realist thinking is retroduction, the process of looking backwards for explanation of how and why things may be. Conducted early in the realist evaluation process, it provides a foundation for evidenced ‘theory gleaning’. Despite retroduction being an inherent part of the realist process, it is often ‘hidden’ in realist reports. This paper explains the thinking behind, alongside an example of, a framework created by the authors to make transparent the retroductive process as used in a realist evaluation of two community End of Life Care services. The approach makes visible the application of the ‘sociological imagination’ and lends robustness to hypotheses by establishing how the authors utilised: wide-ranging potential generative causation; stakeholder and Patient and Public Involvement feedback; literature scoping; and substantive theories at the middle range, specifically Transitions Theory. These stages led to the development of Initial Programme Theories, with a clear history of genesis.

## Introduction

### ‘And all for the want of a horseshoe nail’ (13C proverb)

The proverb ‘For Want of a Nail’ traces a seemingly small and inconsequential (in)action: the loss of a horseshoe nail, through the loss of the horse, the loss of the messenger, the loss of the battle, to the loss of the Kingdom; a significant outcome at first sight not connected to anything as lowly as a horseshoe nail. Researchers employing realist methods would recognise this proverb as a detailed chain of causality. Working backwards from any outcome or phenomenon (in this case, the loss of the Kingdom), through the multiple (in)actions, seeking out structural and agentic cause and constraint, to the identification of the need for a horseshoe nail is any realist researcher’s challenge. What is perhaps less clear is the process utilised by realist researchers to ensure all that goes on in the quest for the detail and description of how, why, when and where the Kingdom came to be lost is transparent.

This paper reports our transparent approach to retroduction in a realist evaluation of two community end of life care (EoLC) services in England, both known as a ‘Rapid Response Service’ (RRS). Retroduction involves looking and thinking backwards, or ‘last things first’; an intentional approach to uncover connections within social systems and experiences (Bhaskar, 1978). As some of the authors were newer to the realist approach than others, a highly transparent approach to the retroduction process was embedded from the study’s start; making visible the early use of the ‘sociological imagination’ (Mills, 1959) when suggesting enablers and barriers to the use of the RRS. Identified by Pawson and Tilley (2004) as the process of ‘thinking it through’, our framework approach deliberately seeks to create a balance between Pawson’s (2006) ‘showing the workings out’ while avoiding pitfalls of over-explanation and recording (Pawson, 2021). This approach generated eight overall areas of enquiry, documented within eight discrete working documents in Microsoft (MS) Word, in a collaborative workspace (MS Teams). As the multiple stages in early development of ideas drew in different forms of searches and knowledge, we sought out potential causal insight; *this creates that* (as opposed to successive causal links, *this follows that*) to produce a series of suppositions, known as Initial Programme Theories (IPTs), that attempt to describe how any programme works, for who it works, why, and in what circumstances (Pawson & Tilley, 2004).

The framework introduced here incorporates malleability across the research cycle; allowing for different ‘nuggets of evidence’ to arise at different junctures (Mirzoev et al., 2020, p. 1244). The initial theory development phase, known as ‘theory gleaning’ (Pawson & Tilley, 2004), gathers a variety of tools and procedures over multiple stages to generate a first full set of suppositions (often called ‘hunches’) of what mechanisms might be occurring, in which contexts, to produce outcomes in the programme(s) under investigation. Once a strong set of these ‘hunches’ has been developed, these suppositions can be rigorously tested in line with data gathered; testing includes whether, and in what ways, they may be correct, how they may be misaligned, or whether there are whole other contexts, mechanisms, and outcomes (CMOs) at work missed by the first stage thinking. As Pawson and Manzano-Santaella (2012, p. 184) explain, ‘a CMO is a proposition and testable one to boot’. Progressively testing CMO’s, provides the opportunity to dig for increasing ‘ontological depth’ (Jagosh, 2020, p. 128). In the final stage, with all data collection, digging, and analysis complete, the bigger picture allows theory consolidation and the development of final agreed Programme Theories (PTs) (Pawson & Tilley, 2004). At the time of writing, the authors have just completed the ‘theory gleaning’ phase and it is that part of the devised framework which is detailed here.

While theory gleaning is an identified phase of realist evaluation, it does not belong solely to this methodological approach; constructing theory, ‘puzzling out’, and problem solving are important aspects of social science inquiry (Mears, 2017; Timmermans & Tavory, 2012). Theory-driven evaluation similarly seeks to identify the principles and design which underpin programmes, and so impact on any implementation, actions, and outcomes; specifically, asking whether the programme achieves what it set out to do (Siebert & Myles, 2019). The sections below present accessible descriptions of how the ‘thinking through’ (Pawson and Tilley, 2004) of the theory gleaning process was actioned and recorded within this realist evaluation, including: research team discussions utilising our ‘sociological imagination’, stakeholder input, Patient and Public Involvement (PPI) representatives’ feedback, literature scoping, and the application of substantive theory at the middle-range. Subsequently, the overall framework demonstrates how these stages can lead to the development of data collection tools; specifically, focus group materials, interview schedules, and a health service use log, to ensure that they reflected and could test the IPTs. Yet it is anticipated that, given the role of theorising and theory building in all forms of qualitative and quantitative inquiry, the stages and approach set out in this article will be of use to researchers within and beyond the realist evaluation community.

## Background

### Theorising and theory

There is much discussion of theory in realist approaches. For those with a sociological background, ‘theory’ and ‘applying theory’, perhaps is most often seen as the act of going to (or coming from) the sociological cannon for grand over-arching abstract theories. Merton’s (1968) ideas of theory construction are embedded in Pawson and Tilley’s (2004) approach, of building explanation onto empirical evidence. When ‘theory’ is discussed here, we are doing so in the following ways.

Realist evaluation is avowedly theory-driven; ‘it searches for and refines explanations of programme effectiveness’ (Pawson & Manzano-Santaella, 2012, p. 178); indeed, ‘programmes are theory incarnate’ (Pawson and Tilley 2004, p. 3). Programmes/interventions under investigation comprise a set of assumptions held by those who designed and deliver them which ‘explain how and why they expect the intervention to reach its objective(s) and in what conditions’ (Marchal et al., 2012, p. 83). Applied to our study, this means that the RRS being evaluated are themselves a representation of a supposition of what is required in such a service and why. We recognise this ‘theory incarnate’ in the service as it stands but, from the start, aimed to produce our own IPTs, creating ‘hunches’ of what works, for who, how, and why.

In the stages delineated below, we demonstrate how we searched for and applied substantive theories at the middle-range. Such mid-range theories (MRTs) are more formal theories that work at a higher level of abstraction than the IPTs and, when applied to the theorising through the realist process, improve external validation (Astbury, 2018). Our work then becomes ‘theoretically transferable’ and our theorising available for testing in different contexts, with different stakeholders’ (Rycroft-Malone et al., 2012, p. 9). Astbury (2018) reminds us of Chen’s (1990) approach of ‘dual theorising’, wherein we theorise from the evidence available to us in the field, and then subsequently, search for formal theory that can provide explanatory value of that evidence. We all theorise, informally and formally; by ensuring we understand and incorporate appropriate abstract, middle-range, and programme theorising, ‘the time needed to develop improvement interventions, optimise their design, [and] and enhance learning from those efforts’ can be shortened (Davidoff et al., 2015, p. 228).

## RAMESES reporting

Guidance exists for the reporting of realist evaluations; the RAMESES Reporting Standards (Greenhalgh et al., 2015; Wong et al., 2016, 2017) were developed to overcome identified flaws in realist reporting. In writing this paper, and in the design and delivery of the research study it discusses, we adhered to these standards. This exposition of our framework is to complement the literature on how to overcome three reporting issues found by the RAMESES project in some earlier realist evaluations (Wong et al., 2016): (i) the importance of identifying causation not correlation; how and why associations occur must be central to findings; (ii) mixed methods are useful to investigate multiple relationships between aspects of context, mechanism, and outcome; and, iii) detailed context-mechanism-outcome configurations (CMO’s) are required in the reporting of findings.

## Method

### Study background

Research identifies that end-of-life care should be person-centered rather than disease specific; but, for patients and their families, there is a chronic under-representation of services to support people as they reach the end-of-life, particularly a shortage of out-of-hours services (Bone et al., 2016; Butler et al., 2018; Clark et al., 2014, Hensen et al, 2016, Robinson et al., 2014). Indeed, a identified the need for significant improvement in community services. Whilst innovative multi-agency community services are emerging, research to date on different service models is limited. One such community service is the Rapid Response Service (RRS) based in primary care; aiming to enable patient choice to maximise their days at home for as long as it feels right, to help reduce pressure on primary health care teams and services, cut the risk of unplanned hospital admissions, and minimise delayed discharges. Yet, there is little UK evidence about the contextual conditions that enable RRS to work to enhance end-of-life experiences and outcomes for patients and their families living in the community nor on the economic impact of the different models of RRS on individuals and society. In addition, the existing evidence about the resource implications of RRS for health and social care providers is sparse, making it particularly difficult for policy makers who are charged with developing agile and responsive services.

This paper describes the IPT development framework created for a two-year evaluation of RSS models in EoLC that sought to investigate what works, for whom and in which circumstances? The research, funded by Marie Curie, considers the effectiveness, costs, and consequences of two different models of specialist palliative care provided to people at the end of life who wish to die at home. Each RSS operates with a different staff skill mix and offers different hours of service. Data will be collected through focus groups, semi-structured interviews, and a health service use log. The study received HRA/REC approval (IRAS ID: 299204) and is portfolio registered (CPMS ID: 50372).

This research uses a realist approach with embedded economic evaluation, applying the notions initially proposed by Anderson and Hardwick (2016). The rationale for integrating the realist and economic evaluation was two-fold. First, to ensure that health economic evidence, concepts, and assumptions were embedded into IPTs from the outset so that they could be tested accordingly. Secondly, to generate a ‘fuller picture’ for the economic analysis, using realist logic to open the ‘black box’ of the RSS ‘intervention’ to identify causation and contextual factors alongside outcomes (Marchal et al., 2012). This ensures all inputs and outputs intrinsic to the implementation and delivery of the intervention are captured. The study is divided into three phases reflecting the structure outlined in the introduction: theory gleaning (phase 1), and theory testing and refining (phase 2 and 3). Phase 1 is the focus of this paper.

## Findings

### Theory gleaning – where to begin?

Theory gleaning in phase 1 was initially generated/conducted through early in-team inclusive and lively discussions, principally from the perspective of the service user. These discussions utilised the ‘sociological imagination’ of all parties but also their academic, clinical, and personal life experiences. Concurrently, we gathered basic information and reflections on the brain-storming developments from our different stakeholder groups, including our PPI representatives in rounds of discussion.

Following Pawson and Tilley’s (2004) advice of ‘thinking things through’ and Pawson's (2006) encouragement to ‘show your workings’, we produced an IPT development framework making visible these early and subsequent stages of theory gleaning. Table S1, available in supplementary files, provides an overview of the five stages undertaken (all recorded in multiple ‘IPT working documents’ held centrally to the research team on MS Teams). The details of each stage are outlined below. An exemplar of a completed IPT working document for IPT1 Communications, with outcomes for all stages set out herein is available as a supplementary file, Exemplar IPT Working Document.

## Stage 1.1: initial brainstorming

Online team brain-storming sessions drove the subsequent identification of eight separate areas of inquiry (see Table S2 in supplementary files for complete list), relying directly on the group application of the ‘sociological imagination’. This ‘imagining’ was produced through active group discussions on MS Teams, using collaborative documents and a group MS OneNote, and incorporated the reflexive thinking of the full diverse research team; clinicians, practitioners, PPI representatives, early career researchers and senior academics in health economics, nursing, and applied health research. When complete, these imaginings were organised into lists of potential barriers and facilitators in each area’s working document; see the Exemplar IPT Working Document in supplementary files for detail on IPT1 Communications.

We drew on Dean’s (2017) conceptualisation of research, which states that research is an interplay of an individual’s biography, their social position, and their social, cultural, and economic resources. As reflexive researchers, we explore our own place ‘within the many contexts, power structures, and identities and subjectivities of the viewpoint’ (Dean, 2017, p. 8). We actively invited this reflexivity, including consideration of our own biographies and experiences and what they could mean for others regarding accessing community end of life services. A team approach, Barry et al. (1999, p. 31) explain, ‘requires both individual and group reflexivity, with a dialogue between the two.’ For another recent detailed approach of early IPT development, see Francis-Auton et al. (2022) explanation of their ‘realist dialogic approach’.

Developing a safe space for honesty, openness, and reflexive sharing in research team discussions requires intentional candid disclosure of experiences and relationships of trust. Discussions about death, dying, and the end of life can be challenging, confronting and emotive; Mannix (2021) however encourages us to not see these conversations as ‘difficult’ but rather as ‘tender’. By following Mannix's (2021) advice, it is possible to provide space for empathetic conversations within teams; listening without judgement, and undertaking what she describes as ‘dancing’, taking turns to lead and participate without pressure to disclose.

### Stage 1.2: stakeholder input

Using the *National Standards for Public Involvement in Research* (NIHR, 2019), PPI was sought in theory gleaning; working with multiple stakeholders, including our PPI representatives, clinicians, and service staff, we adopted a realist logic of analysis to this information gathering, utilising questioning focused on deciphering the how, who, why and what. Feedback and reflection were sought on the early imaginings produced in stage 1.1 and incorporated into short snippets of conversations or bullet points in stage 1.2 section, as illustrated in the Exemplar IPT Working Document in supplementary files. For studies outside of health research, we would still encourage the inclusion of this stage with an appropriate panel of Experts by Experience (EbE) who can provide intimate and insightful real-world reflections (Watson et al., 2022).

### Stage 1.3: grouping of global areas

Both stages 1.1 and 1.2 interrogate multiple potential structural or agentic possibilities, and reason(ing) and resources, of why a service user may (not) use a service offered such as the RRS. As these were recorded and grew, they were slowly grouped together into discrete but related global areas, eventually, identifying eight overall areas (Table S2 in supplementary files sets out the eight areas with a short description of each), all eight have an IPT working document as the exemplar for area 1 provided in the supplementary files. Occasionally, these overlapped, i.e. a potential influence could be applicable to more than one area. Where that occurred, it was embraced; some reasoning and/or resources may be potentially influencing a ‘choice’ to use a service regarding more than one area listed in Table S2. The subsequent stages in this theory gleaning phase (set out in detail below), are designed to interrogate and push further thinking in these areas. With that in mind, keeping these early brain-storming and overall areas as open as possible, encouraged us to hold onto all possibilities and to clearly show those ‘workings out’ and thought processes (Pawson, 2006).

### Stage 2.1: literature

Due to the extensive range of potential sociological, anthropological, psychological, and health economics points of interest identified, two literature reviews were conducted concomitantly. First, a realist scoping review to explore the wider and more global themes from a qualitative perspective. Timeliness and staff restrictions ruled out extensive and systematic literature reviewing. Second, a systematic review of health economic literature was undertaken to capture examples of existing economic evaluations of community or RRS palliative and EoLC services to identify potential costs and consequences/outcomes and measures of these and ensure that these were integrated into the development of the IPT’s. Importantly, as the realist approach is iterative, our literature scoping activity is to be equally iterative and additional literature will be revisited and incorporated over the course of the study.

Main themes arising from the literature and pertinent data were organised into the individual IPT working documents, see the exemplar for area IPT1 Communications in the supplementary files.

### Stage 3.1: if/then/because statements

To extend causal thinking practices further, all stage 1 and 2 insights were reviewed by the research team and if-then-because thinking applied to develop multiple statements per area. We followed Pawson’s (2013, p.xv) well-known explanation of developing real explanatory mechanisms; CCTV cameras are ‘dumb, unthinking bits of glass, metal and electronics [which] actually […] reduce crime through the reasoning of [those] who encountered them’. By adopting this form of theorising, and introducing multiple variables and early casual chains, we distilled and moved the large volume of brain-storming, stakeholder input, and first stage literature scoping and reviewing into formats closer to initial context, mechanism, and outcome configurations (CMOC). The if/then/because statements developed for IPT1 Communications are set out in the exemplar IPT Working Document in the supplementary file.

### Stage 3.2: initial CMOC

As Dalkin et al. (2015), we adopted the CMMO configuration to identify context-mechanism resource-mechanism reasoning-outcome (for a thorough exposition of variations of heuristic configurations, see De Weger et al., 2020). The CMO is the heuristic used throughout realist evaluation to identify the aspects of causality at play and is used to produce ‘plausible explanations of the outcomes observed in the study’ (Marchal et al., 2018, p. 87). At this stage, health economic evidence from the literature, together with health economic concepts of resource use and outcomes and economic assumptions about how resources may impact on the outcomes, were used to develop resource sensitive CMOs and IPTs. As the study progresses, the CMOs will be tested and revised; however, it was useful to begin early probing of the CMOs identified through our brainstorming, stakeholder engagement and literature review ‘gleaning’. Any CMOCs considered or developed were recorded, alongside key points explaining the thinking behind these in the IPT working documents. See those developed for IPT1 Communications in the Exemplar IPT Working Document in supplementary files.

### Stage 4: substantive theories at the middle-range (MRTs)

It is anticipated that different substantive middle-range theories (MRTs) will be investigated and then potentially applied across the eight global areas in forthcoming theory testing and revision phases of the study. Yet, given the importance of MRT to the transferability of any subsequent findings, we undertook early research team discussions and literature scoping to identify possibly useful MRTs before the first set of IPTs were developed. Using the topics ascertained from the eight global areas (see Table S2 in supplementary files for details of each), we searched Google Scholar and reviewed relevant theories. We considered theories of behaviour and implementation such as COM-B (Michie et al., 2011) and Normalisation Process Theory (May et al., 2007) and, although we felt these did not hold the explanatory potential we were looking for at this stage, will likely return to these during further MRT searching later in the research. For a more comprehensive approach to including multiple potential MRTs at this early stage, see Shearn et al.’s (2017) descriptions of the research team’s deliberations and subsequent MRT searches and inclusion.

Transitions Theory (Meleis, 2015) was identified at this stage, given its explanatory potential for understanding change from one state to another. Applicable to several of the eight areas identified, Transitions Theory provides a framework encompassing all the IPTs to some extent and identifies the importance of the experiences of, and response to, change. Furthermore, it provides direction in how to understand these from a service user perspective and an effective service model perspective. Applied to EoLC, it can be used to understand and explain changes in health and illness status, patients and carers’ roles, different sites, and situationally (ibid): all have potential to act as contexts and mechanisms, influencing outcomes. Early team identification of the theories potential suitability and matching is set out in more detail in Box 1.

A matrix was developed in MS Excel to breakdown the theory and question each of the areas. Its use for IPT1 Communications is reproduced as an appendix in MS Word format in (see Table S3 in supplementary files).

### Stage 4.1: IPT

With all the three subsequent stages completed, the research team reconvened and again utilised collaborative software to co-develop a set of eight Initial Programme Theories (IPTs), with some rival theories where appropriate. This incorporated all the earlier stages, including what we had learnt and what we could practically ‘test’ given the study limitations and resources. An important aspect of these IPTs is that they are awaiting ‘testing’ by data collection. They are intended to be ‘initial’ and are malleable; to be revised in subsequent phases. As Pawson and Tilley (1997) explain: the IPT is an extended hypothesis of what might work, for who, in what circumstances. See Exemplar IPT Working Document in supplementary files for IPT1 Communications.

### Stage 5.1: data collection preparation

In realist approaches, as RAMESES reporting standards, the data collection should be driven by the IPT (Greenhalgh et al., 2015; Wong et al., 2016, 2017). Thus, our final task was the fine-tuning of initial data collection tools. This refinement ensured data was collected for each of the eight IPT global areas and that our theories and hypotheses will be ‘tested’ effectively. For the interviews, the schedules will continue to develop iteratively, adapting to learning through concurrent analysis; as appropriate to develop ontological depth (Jagosh, 2020, p. 128), and to search for acuity in causal insight, understanding contextual influence, and reviewing inner mechanisms (Pawson, 2006). By asking ‘how’, ‘who’, ‘when’, ‘what’, and why’, as we dig deeper, we can attempt to understand the key processes in action for participants (Manzano, 2016). We will draw out instances within the interviews where there may be associated costs and benefits and a health service use log has been developed alongside the IPTs to identify costs and benefits associated with (in)formal caring at home at the end of life and contact with other services.

## Discussion

### Making explicit the sociological imagination

The early stages of the approach to theory gleaning, subsequently outlined in the findings, are to make explicit the power and purpose inherent in the adoption of the ‘sociological imagination’ in realist evaluation. By thinking aloud and imagining, then recording our reasoning, we set a broad base to underpin later stages of this phase of realist inquiry. Moving through our identified stages, we demonstrate a process for activating and then evidencing, the early development of CMO’s and IPTs. Other papers have reported their approach to development of IPTs; for example, Fick and Muhajarine (2019) describe working extensively with stakeholders to develop early CMO configurations. Similarly, Griffiths et al. (2022) demonstrate a detailed approach for including stakeholders in the early development stages. Whilst we perhaps provide less in-depth description of our stakeholder involvement, we elucidate a novel and overarching framework of the first phase of realist inquiry - ‘theory gleaning’. Stakeholder inclusion and inputs are less formalised and more iterative in our study, yet still provided insight. As we further develop our approach within the current study and beyond, aspects of both Fick and Muhajarine (2019) and Griffiths et al. (2022) approaches could be added to the ‘stakeholder input’ stage(s).

Through the embedding of literature scoping and reviewing and substantive theory through mid-range theory consideration within the ‘theory gleaning’ phase, we have built rigour into our IPT development; generating deeper understanding, and providing a basis for developing appropriate data collection tools in the subsequent ‘theory testing and revision’ phase of the realist evaluation.

RAMESES II Standards demonstrate how application and integration of theory can and should inform from an early stage, supporting the identification of potential contexts and mechanisms, building links between seemingly unconnected occurrences, and providing powerful explanatory potential (Wong et al., 2016). By considering an identified theory in the earlier stages – Transitions Theory (Meleis, 2015) - we were able to produce tools (see Table S3 in supplementary files) to interrogate and guide thinking about how individuals’ experiences and understandings may impact service use. With these developed, we will revisit throughout the evaluation to enhance understanding and move up and down the ladders of abstraction (Punton et al., 2016; Westhorp, 2012).

### Retroduction

A retroductive approach is employed throughout the stages, demonstrated in the excavation and detection of (hidden) causal mechanisms (Jagosh, 2020, p. 128). Meyer and Lunnay (2013, p 6.1) urge the inclusion of retroduction, alongside abduction in sociological inquiry for rigour, explaining that employing both forms of inference in analysis provides explanatory potential of ‘the social processes that cause events’. This approach looks beyond the (dis)proving of any supposition, or what else might be occurring, to investigating the circumstances within which these events exist. Meyer and Lunnay (2013) adopts the retroductive approach of Danermark et al. (1997) and some aspects of their model can be recognised in our framework; including ‘counterfactual thinking’ and ‘social and thought experiments’. Throughout this initial phase of study, we have continually considered alternatives and constraints: ‘counterfactual thinking requires the researcher to identify the constitutive factors under which concepts exist, and to differentiate between constitutive factors and accidental circumstances’ (Meyer and Lunnay, 2013, p. 2.12). Furthermore, our hypothetical thinking and testing of potential structural and agentic barriers and facilitators through the phases demonstrate our ‘social and thought experiments’.

Retroduction is drawn from critical realist philosophy, Archer et al. (1999, p. 14), identify the major contribution of such a ‘robust stratified ontology […] which doesn’t elide structure and agency, subject and object, voluntarism and determinism, conscious and the unconscious and all the other polarities’. Realist philosophy, and its tool of retroduction, allows investigation of the interplay of structural determinants and our ‘choices’ within those. The stages generated in our framework allowed ‘excavation’ through Bhaskar’s (1978) stratification of reality, through empirical, actual, and into the real.

It is this activity of ontological excavation, finding what is at play behind what we can simply observe, which we have made most visible through developing the approach outlined in this paper. Jagosh (2020, p. 128) explains retroductive thinking inspires the question: ‘how can research distinguish between the intrinsic working of things and the conditional modifications that are always present in manifestation?’. Our multi-staged framework supports the investigation and explication of manifestations of latent mechanisms and generates demonstrable ontological depth. Furthermore, the approach ‘trigger(s) deeply informed thinking necessary for addressing complex entrenched problems’ (Jagosh, 2020, p. 128).

Mukumbang (2021) provides a thorough and detailed explanation of the application of retroduction across the theory gleaning, revision, and consolidation phases of a realist evaluation. In the theory-gleaning phase specifically, Mukumbang explains that we can use retroductive theorising to identify key components of causality. While these cannot include every incidence, they can identify and ‘distil the crucial elements of the process’ (ibid, p.106), therefore providing a generative causation pattern of ‘constrained choice’ (Pawson, 2008, p. 17). As Pawson (2008, p. 13) sets out, ‘causal explanation, in other words, is not a matter of one element (X), or a combination of elements (X1.X2) asserting influence on another (Y), rather it is the association as a whole’. In that sense, demonstrating the multiplicity and complexity of generative causal chains will rarely be as neat as the horseshoe nail metaphor implies and it is not to be considered as an encouragement to follow a linear or successive model of causation. Often, diagrams and maps of casual loops and multiple mechanisms and outcomes can be useful to demonstrate the different components at work. See Mukumbang et al., (2016, 2018) and Mukumbang, (2021) for examples of such tables, figures, and diagrams that grapple with the complexity.

### The avoidance of methodological fetishism

While C W Mills invites us to use our sociological imagination, he insists we do not lose our way and become lost in ‘fetishism’ of methods and approach (also noted by Astbury, 2018, p. 65). Similarly, Pawson and Tilley (2004) encourage us to think clearly and follow the evidence, but avoid any determined or prescriptive formula to using the approach. Although applying retroductive thinking when writing-up our thinking, reflections, and activities into our IPT working documents, through the stages set out above, we recognise that we ‘cannot capture every thought and thoughlet’ (Pawson, 2021, p. 1). Yet we agree with Fick and Muhajarine (2019, p. 553); a lack of guidance on ‘how to’ can ‘create ambiguity and uncertainty’ for those new to realist evaluation. Pawson and Tilley’s early work is discernibly intellectual, leaving a demand for how to ‘operationalise realist methodology’ (Astbury, 2018, p. 64). Our objective here is to elucidate, not further obfuscate, and to avoid an attempt to be dogmatic. We present our approach as *one way* to create transparency and keep thought processes clear, not *the only way*.

## Conclusion: for want of a nail

The approach outlined here is intended to establish and evidence our route through the theory gleaning process, to demonstrate our commitment to the importance of the identification and careful excavation of (potential) underlying mechanisms, within contexts. This is a process of tracing chains of causality, from a seemingly inconsequential (in)action such as the loss of one horseshoe nail to the loss of the Kingdom itself. Mukumbang et al. (2021) reviewed realist studies to explore their utilisation of induction, deduction, abduction, or retroduction. They found a high rate of inconsistency and a significant lack of explanation about *how* analysis occurs and *how* this was appropriate and retroductive, prompting a subsequent paper on the import of retroduction and its inclusion (Mukumbang, 2021). This paper explicates what retroduction *can* look like in a theory gleaning process, what iterative elements it *can* comprise, and how to effectively record this for transparency. The success of this approach to theory gleaning and its application can only be assessed by what follows. That is to say, how well the provisional theories translate into the research design, whether the design proves practicable, if clear findings emerge and whether they have impact. As we approach and assess these through the final stages of the project, we aim to produce subsequent articles to examine the overall effectiveness of the approach once the study is complete.

## Supplementary Material

Supplemental Material

Supplemental Material

## Appendix

**Table:**

**Change is an identifiable in**: curative to palliative to EoL care dying into death and the stages of death (somatic-cellular-post-mortem) social death to bodily death (what Frogatt (2001) calls ‘sequestration’) transition of the space and place of ‘home’ over dying and death period change of caregiver/patient relationship/roles **Managing these changes require differing resources**: physical +/economical +/social +/cultural **The Transitions Theory model includes two phases**: 1. Understanding (the service user) and 2. Intervention (the programme design/strategy). **Phase One- Understanding most appropriate to ‘theory gleaning’ and includes**: change triggers – what kicks off the transition? properties – what are the commonalities of the transition period? conditions – what we are some of the identifiable contexts and resource mechanisms? patterns of response – what are potential reasoning mechanisms and outcomes?

**Table:**

For Want of a Nail *For want of a nail the shoe was lost*. *For want of a shoe the horse was lost*. *For want of a horse the rider was lost*. *For want of a rider the message was lost*. *For want of a message the battle was lost*. *For want of a battle the kingdom was lost*. *And all for the want of a horseshoe nail.*
