# Supplementary material for: ‘For Want of a Nail’: developing a transparent approach to retroduction and early initial programme theory development in a realist evaluation of community end of life care services
Source: Int J Soc Res Methodol. 2023 Mar 12;27(4):417–30. doi: 10.1080/13645579.2023.2184920 (PMC11166047; doi:10.1080/13645579.2023.2184920)
Supplement: Supplemental Material [file TSRM_A_2184920_SM5293.docx]

**Appendix- Supplementary File- TABLE 1**

| **Table 1- Theory Gleaning (Stages 1-5 Overview)** |
| --- |
| **Stage 1**   - 1. Initial Brainstorming – Patients’ Perspective   2. Stakeholder Input   3. Grouping of ‘global areas’ |
| **Stage 2**  2.1 Literature Scoping/Review |
| **Stage 3**  3.1 If/Then/Because Statements  3.2 Initial CMO Configuration |
| **Stage 4**  4.1 Substantive Theories at the Middle-Range (MRTs)  4.2 Revised IPT |
| **Stage 5**  5.1 Data Collection Tools Preparation |

**Appendix – Supplementary Files – TABLE 2**

| **Table 2 – Eight Global Areas** | **Key Point** |
| --- | --- |
| IPT1 Communications | What is the impact of external/internal communications on service users/engagement? |
| IPT2 Values | What are the explicit/implicit values of the RRS and how do these impact on inclusion/exclusion? |
| IPT3 Access | What are the referral routes/gatekeeping/barriers and facilitators? |
| IPT4 Diverse Needs | What is the impact of pain, diagnosis, multi-morbidities, dementia, frailty? |
| IPT5 Geography and Community | Are there urban/rural differences and do community partnerships make a difference? |
| IPT6 24 v 10 hr | How do different service times and the ‘ordeal’ of the night (Sand, 2009) impact service use? |
| IPT7 Timeliness | Responsivity and timeliness (what is ‘rapid’? what is ‘response’?) |
| IPT8 Who (FFC/RRS Staff) | How/are the experiences and resources of friends, family, carers (FFC) in the home already important? +/ How are the competencies/capabilities of the RRS staff important? |

**Appendix – Supplementary Files – TABLE 3**

| **Table 3** Transitions Theory (Melesis, 2014) Matrix for IPT1 Communications |  |
| --- | --- |
| **Transitions Theory (TT) Matrix** | **IPT1 Communications** |
| TT Properties (TRANSITION CONTEXT +/ RESOURCES/RESPONSES) |  |
| Time Span | is information given to those from the beginning to the end of the transition and/or eked out over time? **Does it cover whole period?** Is this all from RRS or from another source? (i.e. could this come pre-RRS from referrer in prep?) |
| Involves Process | as above |
| Disruption | does comms address the loss in transition - **of what was before? i.e. the 3 D's disruption + discontinuity + disconnection?** Does it recognise these will be from other services and other lives and ways of being and address? |
| Awareness Level | does comms take into account the differing awareness levels of patient/caregiver as they enter into the RRS- how so**? i.e. have they been through palliative care in preceding period (weeks/months/year?) and where are they in their awareness that they are in the end of life period and what that means?** |
| Critical Points | does comms consider any **recognised critical points of milestones in the end-of-life period to provide resources, manage expectations: this may happen- this is what we/you can do**? |
| New Skills | Are comms educational or provide advice and guidance or further contacts for support to learn any new skills required? |
| Loss of Familiarity | as 3D's |
| Loss of Support | as 3D's |
| New Support | As ‘New Skills’ |
| Transition Conditions (CONTEXTS +/ PERSONAL RESOURCEs) |  |
| Personal Characteristics | how can a patient or caregivers **personal characteristics be a fit or a barrier to comms?** |
| Perceptions/Meanings of Illness (dying/death) | what are the assumptions in the comms (external/internal) and **do they address how different people perceive death**? |
| Cultural Belief | are comms **culturally appropriate? Are they (unintentionally) exclusive**? |
| Socio-economic status | do comms **provide support and info about how hshd income may impact on ability to be at home and have carers at home at the end of life** inc. equip? heating? Paid time off work? |
| Preparation | are comms **appropriately pitched/recognise the differing material/physical/social/knowledge resources patients/caregivers enter** the RRS **with**? |
| Knowledge | as 'preparation' |
| Environmental Characteristics | as 'preparation'- does it cover different aspects: medical, psychosocial, spiritual, practical. |
| Community Resources | how/do the **comms match/rival community** resources? |
| Societal Conditions | are comms **modified in times of societal change**? i.e. covid-19? |
| **Nursing Therapeutics (PROGRAMME RESOURCES)** |  |
| Readiness and Risk | how in comms pre/with newly referred **are their readiness and risks assessed**? |
| Transition Preparation | do comms **prepare for all aspects of transition to come?** How so? Why not? (i.e. palliative-EoL; EoL-death-post) |
| Role Support | do comms **recognise and support the potential changing of roles of patient/caregiver** (i.e. partner to caregiver or cared for to carer?) |
| Creating Healthy Environment | in what way(s) **do the comms provided contribute to a healthy environment** around the patient? |
| Monitoring | how does the comms **(written/spoken/resources as well as (un)planned contact) work to support monitoring of patients through RRS and how does this work in collaboration with other services**? |
| Debriefing | how are **comms used to manage debriefing** - post-events and post-death? |
| Response Patterns (RESPONSES + OUTCOMES) |  |
| **Process Indicators** |  |
| Feeling Connected: | how/does RRS comms lead to service users feeling more connected? |
| Interacting: | how/does RRS comms lead to service users improved interaction? |
| Location and being Situated: | how/does RRS comms lead to service users feeling they are where they |
