# Supplementary material for: ‘For Want of a Nail’: developing a transparent approach to retroduction and early initial programme theory development in a realist evaluation of community end of life care services
Source: Int J Soc Res Methodol. 2023 Mar 12;27(4):417–30. doi: 10.1080/13645579.2023.2184920 (PMC11166047; doi:10.1080/13645579.2023.2184920)
Supplement: Supplemental Material [file TSRM_A_2184920_SM5292.docx]

**Appendix – Supplementary Files – EXEMPLAR IPT WORKING DOCUMENT: IPT 1 Communications**

| **Exemplar RRS Project IPT Working Document – Theory Gleaning Stages 1-5**  **IPT1 Communications** |
| --- |
| **Stage 1**  **1.1 Brain-Storming**   1. I am **unaware** of the rapid response service (RRS). 2. I am aware of the RRS but I am not sure about **when and/or why I should contact** them: I don't understand what the service can offer me e.g., what they offer, how and when (10 vs 24 hour, during the day or night?)? Is it straightforward to contact them (especially in a crisis) and/or when do I contact my own General Practitioner (GP)/District Nurse (DN)/111 or 999 emergency response? 3. I do not know if this is the **right service** for us - Am I confident that they can provide the care I need and/or when I need it: e.g., are there any communications that help manage my expectations of the RRS and what form do these come in (i.e., conversations, leaflets, directed to a website)? e.g., have communications about end of life occurred - are the patient/carer/other HSCPs involved in care and decision making agreed/aware that I/we are at end of life and it is appropriate for me to receive end of life care? 4. **Some symptoms are difficult to manage**at home for me as a carer e.g., cannot keep patient dry, cannot get rest overnight, cannot drive patients into hospital, will the RRS support these needs, or will we need to go to hospital/district nurses for care? 5. Will the RRS **co-ordinate with other healthcare professionals** involved in my care? will RRS communicate directly with each other? do I need to communicate with them separately? 6. **I do not want to contact the RRS**: I already have a trusting relationship with another service/have a preference for another team or professional 7. I am **not able to communicate** with RRS/GP/DN: am I being heard? I am too tired to communicate what I need and/or do I hear (retain) what is being shared? 8. English is not my **first language** – is information available in different languages?    1. **Stakeholder Input**   Questions  What is the RRS?  How is it supposed to work? How does it work?  Who does it work best for and why?  Who does it work least for and why?  Who are all the stakeholders (who is/was involved)?  What/who gets in the way of it working (policy/procedure)?  What/who supports it working?  Findings (sub-headings)  Difficult communications in frightening/chaotic times (adaptability/flexibility)  Missing communications (referral partners and/or service users unaware or barrier)  Differing communications between service and service users (whose conversations/what conversations/when?)  Research Team Discussion  There are a few forms of communication that could be discussed and confused:   1. RRS internal communications- between the service and their staff 2. RRS external communications– to DNs/GPs/Emergency Services/Discharge Teams/Hospices/General Public 3. RRS communications to patients/FFC explanation of what service is and when to use   RRS communications to patients/caregivers about when to use them and when to use other services and what can expect at end of life? |
| **Stage 2**  **2.1 Literature Scoping/Review**  Identified themes (sub-headings):   - Guidelines, policy, and practice - Equity, inclusion, and communications - Time, resources, and opportunity for communications - Sharing information - Planning and initiation, who, how, when – responsibility - Denial and acceptance- coping strategies - Awareness of death and dying/death literacy/hidden death - Awareness and reassurance - Knowledge (autonomy/control) - The economics of hope |
| **Stage 3**  **3.1: If/Then/Because Statements**   1. **If**patients/carers are aware of what the RRS can offer, **then** they will be able to make an informed decision to use the service, **because** they will have the appropriate information available to them 2. **If**RRS's clearly communicate to patients/carers what services they provide, when and how, **then** patients/carers will feel confident to know what to ask for, **because**they are equipped with the appropriate expectations of the service 3. **If**RRS's demonstrate that they co-ordinate well with other services, **then**patients/carers will feel greater reassurance in using the service with the knowledge that their care is being streamlined/ consistent, **because** the necessary clear pathways between services are in place 4. **If** RRS staff communicate appropriately/effectively with patients/carers when they attend, **then** patients/carers will be likely to use the service again in times of need **because** they trust the service/have built a relationship with staff 5. **If**RRS's demonstrate that patients/carers are being listened to, **then**patients/carers will place trust in use the service to meet their individual needs,**because**they feel part of decision-making and have more control over their care 6. **If**information about death and dying is provided by RRS's to patients/carers, **then**they will feel confident about using the service, **because** they know what to expect 7. **If**information about the service is available in different languages or using interpreters, **then** patients/carers will feel reassured that the service is right for their needs, **because** they understand what the service offers   **3.2 Initial CMO Configuration**  **CONTEXT:** Patients and their friends, families, and carers (FFC) are aware of the RRS  **MECHANISM-RESOURCE**: open, appropriate, and timely communication is accessed/accessible  **MECHANISM- REASONING/RESPONSE**: patients and FFC understand the service and more about death and dying, feel reassured, and can make an informed decision about death and dying at home  **OUTCOME**: the RRS can be accessed as and when appropriate and the patient can die at home as their choice |
| **Stage 4**  **4.1 Substantive Theories at the Middle-Range (MRTs)**  Transitions Theory- see reproduced matrix in table 3 supplementary files for application.  **4.2 Revised IPT**  If patients and their families have the opportunity to receive open, (honest?), and timely communication **(context)** about the RRS at the end of life (what it is, what it offers, when and why) **(mechanism-resource)**, they can make an informed decision about death and dying at home **(mechanism-reasoning)** and so will self-refer to the RRS **(outcome-1)**, and/or post-referral (self or DN/GP) use the RRS, as opposed to another emergency service, in times of need **(outcome-2)**, and admissions to hospital at end of life will be reduced **(outcome-3)** |
| **Stage 5**  **5.1 Data Collection Tools Preparation**  **Group 1 – RRS Staff**  Communications  *Honest, open, and timely communication between RRS staff and service users supports death at home as people understand what to expect.*  *Is that your experience?*  Prompts:   1. Who is responsible for communication around death, dying, and bereavement? (the RRS, other services, both and all?) What function does it perform? 2. What should this communication look like from an RRS service – time, location, materials? 3. Should it cover what RRS does, how, when, and why and when to call another service? Should it cover contacting other services on the service users behalf? What differences does that make?   **Group 2-4 – External Staff, Carers, and Patients**  For iterative development. |
